# Oculomotor Remapping of Visual Information to Foveal Retinotopic Cortex

**DOI:** 10.3389/fnsys.2016.00054

**Published:** 2016-06-21

**Authors:** Tomas Knapen, Jascha D. Swisher, Frank Tong, Patrick Cavanagh

**Affiliations:** ^1^Cognitive Psychology, Vrije Universiteit AmsterdamAmsterdam, Netherlands; ^2^Brain and Cognition, University of AmsterdamAmsterdam, Netherlands; ^3^Laboratoire Psychologie de la Perception, Université Paris DescartesParis, France; ^4^Department of Psychology, Vanderbilt UniversityNashville, TN, USA; ^5^Department of Psychological and Brain Research, Dartmouth CollegeHanover, NH, USA

**Keywords:** fMRI, saccades, remapping, retinotopic mapping, visual cortex

## Abstract

Our eyes continually jump around the visual scene to bring the high-resolution, central part of our vision onto objects of interest. We are oblivious to these abrupt shifts, perceiving the visual world to appear reassuringly stable. A process called remapping has been proposed to mediate this perceptual stability for attended objects by shifting their retinotopic representation to compensate for the effects of the upcoming eye movement. In everyday vision, observers make goal-directed eye movements towards items of interest bringing them to the fovea and, for these items, the remapped activity should impinge on foveal regions of the retinotopic maps in visual cortex. Previous research has focused instead on remapping for targets that were not saccade goals, where activity is remapped to a new peripheral location rather than to the foveal representation. We used functional magnetic resonance imaging (fMRI) and a phase-encoding design to investigate remapping of spatial patterns of activity towards the fovea/parafovea for saccade targets that were removed prior to completion of the eye movement. We found strong evidence of foveal remapping in retinotopic visual areas, which failed to occur when observers merely attended to the same peripheral target without making eye movements towards it. Significantly, the spatial profile of the remapped response matched the orientation and size of the saccade target, and was appropriately scaled to reflect the retinal extent of the stimulus had it been foveated. We conclude that this remapping of spatially structured information to the fovea may serve as an important mechanism to support our world-centered sense of location across goal-directed eye movements under natural viewing conditions.

## Introduction

We scan the world around us with frequent eye movements that bring items of interest from the periphery into the fovea where vision is most acute (Buswell, 1935). However, we are usually unaware of these profound changes in retinal stimulation, and instead perceive the scene around us as stable. This impression of stability depends strongly on brain processes that integrate information about current eye position (Merriam et al., 2013; Connolly et al., 2015; Strappini et al., 2015) and upcoming eye movement plans to update retinotopic visual processing (Wurtz, 2008). According to one such process, called perisaccadic remapping, visual neurons increase their activity around and even before the time that an upcoming saccade will bring a stimulus into their receptive fields (Duhamel et al., 1992; Nakamura and Colby, 2002). Recent findings in V4 show that this remapping response is seen in the earlier activity (Neupane et al., 2016) whereas later activity shows an activity shift toward the saccade target (see also, Zirnsak et al., 2014, for area frontal eye fields, FEF). Behavioral results have indicated that perisaccadic remapping is a spatially precise process (Collins et al., 2009; Szinte and Cavanagh, 2011), but the neural mechanisms underlying this precision are not well understood.

**Figure 1:**
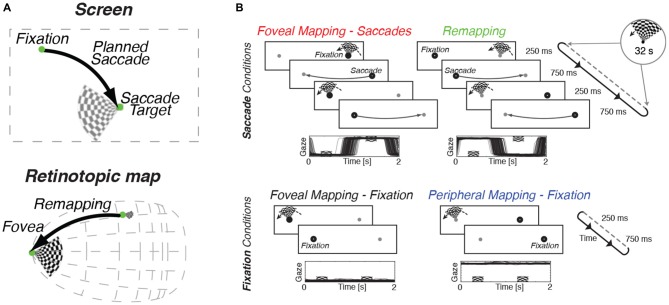
**Design of experimental task and oculomotor results. (A)** When preparing an eye movement to an item of interest, the motor plan for the upcoming saccade is incorporated into visual processing by a process called remapping that transfers target-related activity to the expected post-saccadic location.** (B)** In all conditions, a slowly rotating wedge stimulus was used to evoke a periodic pattern of stimulation in retinotopic cortex. Subjects performed a color-change detection task on the wedge in all conditions. Two fixation marks, one on either side of the screen, were presented throughout the experimental runs. Eye-position traces for one representative subject are shown below the trial sequence for all conditions. Top row: Saccade conditions. Subjects made back-and-forth saccades between the fixation marks at a rate of 1 Hz. This pattern of eye movements ensured that the subject was looking at the stimulus center as it was shown (Foveal Mapping—Saccades). In the Remapping condition, the rotating wedge stimulus was shown not at fixation but at the target of the upcoming eye movement. This ensured that remapping responses would be directed towards the fovea while the stimulus was shown peripherally. Bottom row: Fixation control conditions. Subjects fixated one side of the screen, and stimuli were shown either at fixation (Foveal Mapping) or in the periphery (Peripheral Mapping).

In everyday life, we make saccades toward items of interest to bring them to the fovea as depicted in Figure 1A, left. Thus, under normal viewing conditions, predictive remapping responses should predominantly affect foveal regions of the brain’s retinotopic areas where the saccade goal is expected to land (Figure 1A, right). Indeed, there is behavioral and single-unit evidence that suggests that visual representations are attracted to the saccade target around the time of an eye movement (Deubel and Schneider, 1996; Zirnsak et al., 2014). As the fovea is the location on the retinotopic map that the saccade target will occupy after the saccade, this underlines the importance of the more foveal regions on the retinotopic map during oculomotor behavior. Previous neuroimaging studies have investigated the remapping of stimuli from one peripheral location to another. These studies find that the remapping of a peripheral target leads to strong responses in the parietal lobe (Merriam et al., 2003), and weaker effects in retinotopic visual areas (Merriam et al., 2007). Here we investigated the characteristics of remapping responses to foveal rather than peripheral locations. Specifically, we used functional magnetic resonance imaging (fMRI) to measure the remapped activity for the saccade targets themselves, activity that should impinge on near-foveal regions of retinotopic visual cortex. As in the earlier studies, we removed the target prior to the eye movement. Moreover, our targets had a distinctive wedge shape that varied in orientation over time, which allowed us to test whether remapping of activation to the foveal region would preserve properties of the spatial configuration of the target.

We used a slowly rotating wedge stimulus, as has been used to measure preferred angular position in standard retinotopic mapping (Engel et al., 1994; Sereno et al., 1994, 1995; Larsson and Heeger, 2006; Swisher et al., 2007). This design allowed us to translate spatial information into temporal information, namely the phase of periodic responses. The wedge stimulus consisted of a contrast-reversing checkerboard that counterphased at 8 Hz, and rotated in steps around the apex of the wedge (see Figure 1). Every second, the wedge was presented briefly (250 ms) and then removed (750 ms), after which it reappeared rotated by 11.25° around its apex; this led to one full rotation every 32 s. In the “foveal mapping—fixation” condition, the wedge was displayed with its apex directly at fixation and no saccades were made. In the “remapping” condition, observers made back-and-forth saccades between two fixation markers on either side of the screen, and the wedge was always presented at the marker location that was not being fixated. The wedge was presented peripherally immediately before a saccade was directed to the fixation mark at its apex, a condition designed to evoke a remapping response with each saccade, that cycled back and forth at 1 Hz (see Figure 1B for details). Critically, in the remapping condition, the wedge was extinguished before the saccade was completed, so that the region around the fovea was never directly stimulated by visual input. Once the saccade had landed, the slightly rotated wedge reappeared at its new location on the opposite side of the screen and a subsequent saccade was planned to the new wedge location (Figure 1B). This experimental design allowed us to test whether systematic remapping occurs around the foveal representation of retinotopic visual cortex, since the wedge changes position over time, relative to the saccade target (i.e., apex), yet is removed prior to saccade completion. If predictive retinotopic remapping occurs during this experiment, we should observe similar temporal phases of BOLD responses in the foveal retinotopic visual cortex as those that are evoked by direct foveal mapping. If no remapping occurs, then there should be no systematic relationship between the phases of responses to direct viewing and remapping conditions.

In addition to these two key conditions, two additional control conditions were examined. In the foveal mapping-saccades condition, participants made an eye movement between each stimulus presentation, but here, the wedge stimulus was presented after completion of the eye movement. This control condition allowed us to map the foveal representation and to obtain phase estimates that accounted for any additional noise resulting from performance of the saccade task. This “foveal mapping—saccade” condition differs from the primary remapping condition only in the relative timing of the eye movements and stimulus presentation (Figure 1B). In the final control condition, participants had to maintain stable fixation throughout the run while attending to the wedge stimulus presented in the periphery. The retinal stimulation in this “peripheral mapping—fixation” condition was expected to be comparable to that in the remapping condition, but since no eye movements occurred, no remapping response was expected.

In all conditions, observers performed a color change detection task on the rotating wedge stimulus, a task they had mastered in pre-scanning training sessions. The difficulty of the color task was titrated such that it engaged observers’ attention but did not hamper their ability to comply with the instructed eye movement task.

## Materials and Methods

### Stimuli

Stimuli were 90° angular wedges filled with a circular checkerboard pattern that counterphased at 8 Hz. The wedges subtended 3.5° of visual angle, and rotated counterclockwise by 11.25° every second, completing a full rotation every 32 s. Saccade targets were placed at the center of both sides of the screen, separated by a distance of 14°. Stimulus duration was 250 ms, with a 750 ms blank period, while the two fixation markers remained continuously visible. In all conditions, the fixation mark would change color from black to white 250 ms after stimulus offset; this served as a cue for the observer to make a saccade (in the saccade experimental conditions only). This delay was chosen in pilot experiments to minimize the amount of eye movement errors observers made. For the peripheral mapping conditions, fixation was on one side of the screen with stimulation on the other for an entire run, with runs of either side run performed by all subjects but one. The data from these separate runs were combined per hemisphere contralateral to visual stimulation. On half of the trials, chosen randomly, the wedge would change color to blue-yellow or red-green hue combinations. Subjects pressed a button to indicate when this happened. All button presses registered within 1 s of the end of stimulus presentation were used in the calculation of d’.

### Imaging

Scanning was performed at two separate locations, Vanderbilt University Institute for Imaging Science (VUIIS) in Nashville, TN, USA and Spinoza Center, University of Amsterdam, Amsterdam, Netherlands, on 3 Tesla Philips Achieva scanners, identical with the exception of MRI coil and eye tracking/display arrangement. All experimental procedures were in accordance with the Helsinki treaty and approved by each institute’s institutional review board. Subjects gave written informed consent before scanning commenced. All functional scans were T2* weighted EPI images and gathered with 28 slices oriented perpendicular to the calcarine sulcus, covering occipital and posterior parietal regions of the brain. TR was 2 s, TE 27.6 ms and flip angle was 76.1° with a voxel size of 3 × 3 × 3 mm, duration of each scan was 4.5 min. These scans were coregistered to a 1 × 1 × 1 mm 3DTFE T1 anatomical scan which was segmented and inflated using FreeSurfer tools and acquired in a different session.

At Vanderbilt University, an 8-channel head coil was used, and stimuli were projected by an Eiki LC-X60 LCD projector with a Navitar zoom lens (60 Hz, 25 ms luminance fall time) onto a screen inside the scanner bore while eye movements were recorded at 60 Hz by an Applied Science Laboratories EYE-TRAC 6 eye-tracking system. To stabilize the subjects’ head, a bitebar system was used at the VUIIS. Four male subjects, aged 27–31, two of whom were authors, were scanned at VUIIS. Each completed at least four runs in each of the experimental conditions in a single session. Spillover time at the end of the session was allocated to performing additional remapping runs, of which there were on average 5.75 per subject.

At the University of Amsterdam, three female subjects, aged 25–30 were scanned. Each completed at least two runs in each of the conditions in a single session. Again, spillover time at the end of the session was allocated to additional remapping runs, of which an average of 5.3 were collected per subject. A 32-channel head coil was used, and stimuli were projected onto a screen just outside the scanner bore by a ProjectionDesign F22 DLP projector (60 Hz, <1 ms luminance decay time) while eye movements were recorded at 2 kHz by an SR Research Eyelink eye tracker. There are no qualitative differences between the data from the two different research centers (Supplementary Figure). We conclude that differences between the experiments in both scanning facilities, and notably screen persistence, did not materially affect our results. All subjects had normal or corrected-to-normal visual acuity.

### Data Analysis fMRI

Data were registered to the anatomical T1 volume using the bbregister (FreeSurfer) program after which registration was verified by eye and was adjusted by hand when needed. Motion correction was performed on the functional data using mcflirt (FMRIB’s Software Library, FSL), and the first cycle of each run was discarded. Retinotopic mapping for the delineation of visual areas was performed in a separate scanning session, based on standard phase-encoded procedures (Sereno et al., 1995; Swisher et al., 2007). Phase-encoded data analysis for the main experiment was done in GNU octave by performing linear trend subtraction per run, followed by Fast Fourier Transform applied to concatenated voxel timecourses for all runs in each condition and analyzing the Fourier magnitude and phase at the stimulus frequency. *P*-values for each voxel were calculated by dividing the Fourier power at the stimulus frequency with that at surrounding frequencies and performing an *F*-test on this ratio, with the number of TRs as degrees of freedom. The *p*-values were then used for thresholding of the phase images (Figure 3). All further data analysis was conducted in python, using numpy and scipy libraries.

**Figure 2:**
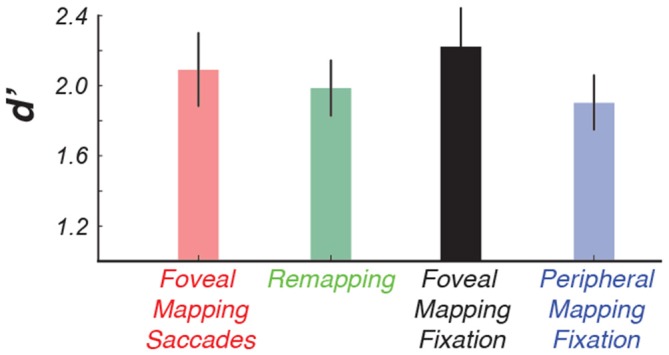
**The results of the color-change detection task indicate that making intermittent saccades did not cause a decline in performance.** This allowed us to directly compare conditions that had identical retinal stimulation, that is, use the peripheral mapping condition as a control condition for our remapping condition.

**Figure 3:**
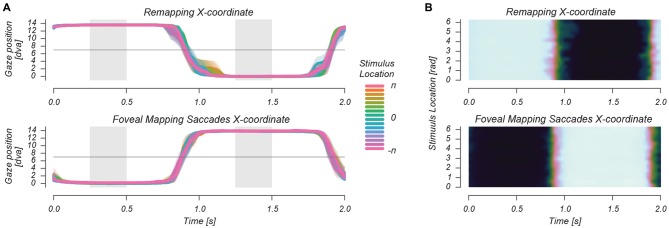
**Horizontal eye traces during a 2 s repetition time (TR) in two separate representations (A,B).** In the Remapping and Foveal mapping—Saccades conditions, observers made saccades at 1 Hz. These are depicted for each of the phases/locations of the stimulus as it circled the saccade target at 1/32 Hz. This figure shows that there were no differences in stimulus-location locked saccade timing, landing position and fixation location during stimulus presentation between the two saccade conditions.

**Figure 4:**
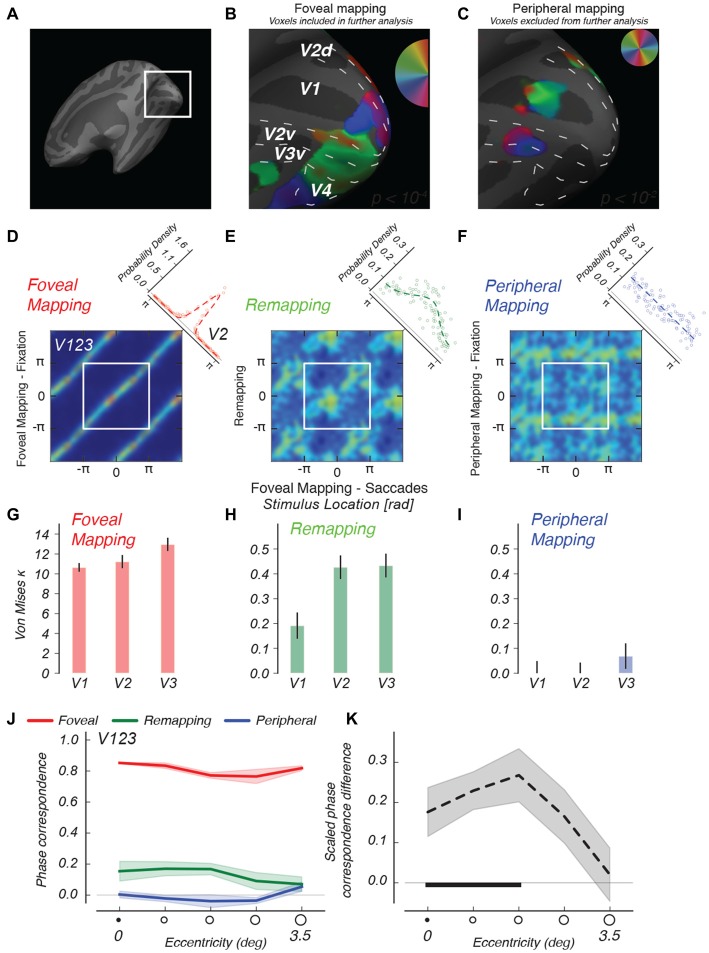
**Descriptive functional imaging results. (A)** Detailed view of retinotopic visual cortex on inflated right hemisphere of an example subject.** (B)** Regions stimulated in the case of foveal mapping, fixation, are used to select voxels for further analysis.** (C)** Regions stimulated in the case of peripheral mapping are selected using a more lenient criterion and used to conservatively exclude voxels from further analysis.** (D–F)** 2d-histograms comparing phase values in different conditions, for V1 through V3, averaged across observers. Comparison of phase estimates between foveal mapping conditions with and without saccades** (D)** shows phase correspondence due to identical visual stimulation as diagonal bands. The diagonal inset depicts the distribution of phase differences between conditions over voxels, and the best-fitting von Mises function to that distribution.** (E)** Comparing remapping and foveal mapping shows similar, if less strong, banding, indicating the remapping to foveal regions of shape information that follows the same phase advance as that measured for the foveal stimulus.** (F)** No diagonal banding indicative of shape information is found when the rotating wedge stimulus is attended but no eye movements are made. For these 2d-histograms, color scales were adjusted per condition.** (G–I)** Kappa parameters of the best-fitting von Mises distributions for different visual areas. In V1 through V3, the peripheral condition (blue) remains similar to a uniform distribution. Error bars represent fit parameter estimate variance.** (J)** We ordered all voxels according to their eccentricity and analyzed the phase correspondence (see “Materials and Methods” Section) in all three conditions.** (K)** We calculated the phase correspondence index between Peripheral and Remapping conditions, and divided by the Foveal condition’s phase correspondence value. This analysis shows that shape-specific remapping responses converge specifically onto the more foveal regions of retinotopic cortex. The horizontal black bar indicates eccentricities at which remapping phase correspondence is significantly greater (*p* < 0.05) than in the fixation control condition. Shaded regions indicate SEM across subjects.

We analyzed phase estimates for all voxels in visual areas V1–V3 in two ways, both relying on the per-voxel phase preference estimates for each of the four conditions. The first analysis entered on the per-voxel difference in phase estimates between conditions, as this measure quantifies how well the periodic signals in those voxel correspond in phase. In each retinotopic area, these phase differences form a distribution that is centered around zero and whose width quantifies the amount of phase correspondence (see Figure 4). We fit von Mises distributions (a circular analog of the Gaussian distribution) to the phase differences between conditions. To ensure comparability across participant, we created histograms of the phase differences for each participant 96 bins and normalized by dividing the histogram values by the amount of voxels in that region. This division ensures that subjects are weighted equally in this analysis, even though visual areas comprise different numbers of voxels in different participant. We performed least-squares regression while keeping the mean of the distribution fixed at 0, to specifically test the width of the von Mises distribution. Permutation tests on these data were conducted by shuffling, within a participant, the condition labels for voxels within each region of interest. Thereafter, the data were binned, normalized, and averaged across participant to provide a null distribution against which we tested the dispersion/kappa values in the original data. 2d-histograms in Figure 4 result from 2d-histograms, averaged across participant after dividing by the amount of voxels in that region for that participant. In a second analysis based on von Mises distribution fits, the mean parameter of the distribution was also allowed to vary. For this analysis, we fit the data from each observer separately, without binning across voxels, using a maximum likelihood procedure. This was bootstrapped 1000 times for each subject (Supplementary Figure shows scatter plots of these fitting procedure results).

The mean phase difference measures reported in the Supplementary Figure, as well as the results of the separate analysis for foveal and more eccentric regions are the mean of the absolute value of the phase differences between two conditions, which provides a model-free estimate of the phase difference spread. This value is transformed according to

$$\left(1-\frac{\bar{|\phi_{1}-\phi_{2}|}}{\pi }\right),$$

so that a value of 0 indicates no phase correspondence, and a value of 1 indicates perfect phase correspondence.

In the MVPA analysis, we used the per-voxel phase preference estimates from the saccade-foveal stimulation mapping condition as a forward model of visual location selectivity. This forward model allowed per-TR decoding of the location being represented in the pattern of activation across a visual region. We conducted the analysis as follows. Phase preferences of a set of voxels were represented as a vector of complex numbers, one unit-length complex number per voxel, of which the angle is the phase preference of that voxel. These unit-length complex numbers were then scaled by multiplying them by the F-statistic of each voxel, a step which creates a weighting amplifying the influence of more reliable voxels in subsequent steps.

We calculated the dot product of these scaled complex numbers and the *Z*-scored BOLD values of each voxel in a region, for each of the three remaining conditions. This provides us with a single complex-number estimate of the location information represented in a given region during each TR. These complex-number time-courses were *Z*-scored over time to prevent signal amplitude differences between conditions from impacting our results. Because our design was periodic, we averaged the complex time-courses across 32 s/16 TR cycles, and took the angle to denote “represented stimulus phase”. For single-value quantification of this full-period time course, we quantified the strength of an area’s representation of visual location by linearly projecting the profiles for remapping and fixation-periphery control conditions onto the foveal fixation mapping condition according to

$$X_{\text{p}}\;=\;\frac{X\cdot FM}{\left\|FM\right\|}$$

where *X* is the time-course for each condition, and FM is the Foveal Mapping condition’s time course. These projections, and the differences between them, were tested using one-sample *t*-tests.

### Data Analysis Eye Movement/Position

Eye position signals from both ASL and EyeLink eye trackers were resampled to 60 Hz. Then, the stimulus cycle was divided into 16 periods of 2.0 s TR duration, during which one back-and-forth saccade pattern was performed. Gaze position within these 2 s intervals was averaged across all TRs at a given phase of the stimulus, in order to investigate whether stimulus location influenced saccadic behavior. Measures of this oculomotor behavior (gaze during stimulus presentation, saccade landing point, and saccade latency) were tested using repeated measures ANOVA with factors “stimulus phase” and “condition” using the aov package in R.

## Results

### Behavioral Results

Making saccades did not affect observers’ ability to report brief changes in the color of the wedge stimulus (*p* > 0.75), although performance was generally better when the wedge stimulus appeared near the fovea, as compared to the far periphery (2-way repeated measures analysis of variance (ANOVA; retinotopic position × saccades), *F* = 7.73, *p* < 0.01, see Figure 2).

### Oculomotor Results

To assure that no visual stimulation of foveal retinotopic regions occurred in the remapping condition, we only analyzed functional imaging data from Remapping runs in which the retinal region that was stimulated during the “Fixation Mapping” condition was never presented with the peripheral visual stimulus. Thus we rejected 29% of the runs in which early, late or failed saccades caused retinal stimulation of the retinotopic region of interest (ROI). We further analyzed whether saccade timing, saccade landing position and gaze position during stimulus presentation differed between remapping and foveal saccade mapping conditions. Figure 3 shows the average horizontal gaze position during the 2 s back-and-forth saccade pattern, for different stimulus locations around the fixation mark (denoted as *stimulus phase*). We found no systematic differences in saccade landing location and gaze position during stimulus presentation between the remapping and foveal mapping conditions, as tested using a repeated measures ANOVA (all *p* for condition and condition × stimulus phase > 0.6). There was furthermore no significant difference between conditions in average saccade latency, defined as the timepoint at which gaze position crossed the screen’s vertical meridian, all *p* > 0.5.

### Neuroimaging Results

For the analysis of imaging data, we used the fixation control conditions to identify retinotopic regions responding to foveal and peripheral visual stimulation. In our analyses, we specifically used voxels responsive in the Foveal Mapping condition and unresponsive in the Peripheral Mapping condition (see Figures 4A–C). We constructed foveal ROIs by using a conservative statistical threshold (*p* < 10^−4^) for the foveal mapping condition (Figure 4B) and subsequently excluded from this ROI any voxels that responded to peripheral stimulation defined using a more lenient threshold (*p* < 10^−2^, see Figure 4C, less than 10% overlap between voxel populations). We note that the region on the retinotopic map that represents the most central region of the visual field, near the fixation mark, is blank in this figure. It is likely that small fixation errors cause the fixation mark to strongly stimulate this retinotopic region, causing the strength of the phase-encoded signal at 1/32 Hz to wane (Schira et al., 2009).

Our aim was to investigate whether information pertaining to the spatial position of the wedge stimulus, relative to the saccade target, was remapped to the foveal areas of retinotopic cortex. We investigated this using two separate analyses. The first analysis centers on the location preferences of voxels as found in a standard phase-encoded retinotopic mapping analysis, which we compared across conditions to describe of our results. The second analysis used voxels’ location preferences from the saccade-foveal mapping condition as a forward model to decode location information at each fMRI time point from visual ROIs.

### Phase Preference Characterization

Remapped location information should, in our periodic phase-encoded design, appear as consistent phase preferences for direct visual stimulation and remapping conditions on a voxel by voxel basis. To visualize the phase relations between conditions, we constructed two-dimensional histograms of voxels’ phase preference from areas V1–V3 that share the first cortical foveal confluence (Schira et al., 2009), and averaged these data across participant after dividing these histograms by the number of voxels in each participant. We first validated our procedure with foveal stimulation by means of the stepping wedge fixated at its apex. We tested whether making saccades affected voxels’ location preference, as quantified by preferred phase, by comparing conditions with identical foveal stimuli, with and without saccades (Figure 4D, left). We found a very strong correspondence between the phase estimates in the two conditions as evidenced by the strong diagonal banding in the histogram across the full range of phase preferences. This pattern indicates that performance of the saccade task had minimal impact on the pattern or quality of retinotopic maps in the foveal visual cortex. Furthermore, this level of phase correspondence aids our interpretation of subsequent comparisons by providing an upper bound for the phase correspondence in the other conditions. One way of quantifying phase relations between conditions is to take the circular phase difference between conditions for each voxel, which should be distributed according to a circular von Mises distribution (diagonal inset, Figure 4D).

We then used von Mises fits, which parameterize the peakedness of the phase-difference distribution, to characterize remapped activations. We predicted a similarity between response phases in the “foveal mapping—saccades” condition and the remapping condition, as remapped activations should correspond spatially to visual stimulation. Indeed, Figure 4E, center, shows visible diagonal banding in the Remapping condition similar to the Foveal Mapping case. Indeed, the von Mises function fit to the phase difference distribution is decidedly peaked (diagonal inset). This structure indicates that foveal-parafoveal regions in early visual areas that were never visually stimulated nevertheless represent information about the spatial configuration of the saccade target, a notion we explicitly test in subsequent analyses.

We also compared the activation for foveal stimulation to that observed in the peripheral stimulation control condition. In this control experiment, no remapping of retinotopic responses was expected because saccades were never directed toward the peripheral stimulus. Figure 4F, right, shows no clear phase relationship between these two conditions in areas V1–V3 during steady fixation, and von Mises fits describe a distribution that is close to uniform. This means that, without eye movements, there was no correspondence between the pattern of retinotopic responses for foveal and peripheral stimulation. Thus, the foveally remapped signals we observed in the remapping condition cannot be attributed simply to covert attention directed to a rotating stimulus in the periphery. The overall pattern of results indicates that phase correspondence in the remapping condition is a true remapped signal, as it depends critically on the saccades made by the observer.

We performed fits of the von Mises function to the phase difference distributions while keeping the mean of the distribution fixed at 0. This allowed us to quantify the spread of the phase differences between conditions over and above the descriptive patterns observed above. The kappa parameter of the von Mises distribution is commensurate with the reciprocal of the standard deviation of a normal distribution, with 0 indicating a uniform distribution and higher values indicating a more peaked distribution. Analyzing these phase differences from foveal regions in separate visual areas, we find that phase differences are very peaked in the foveal stimulation conditions, indicating very strong phase correspondence between visual mapping conditions with and without saccades (Figure 4G). For all visual areas analyzed here, the kappa parameters were greater in the remapping condition than in the condition with the identical peripheral stimulus but no saccades (all *p* < 10^−4^, within-subject permutation test, see “Materials and Methods” Section), indicating that these remapped signals depended on the execution of saccades and could not be explained solely in terms of the bottom-up stimulus input or by top-down spatial attention directed to a peripheral stimulus without making saccades. We also fitted these data by letting both the mean and the kappa parameters of the von Mises distribution vary. The results of this procedure indicated that the remapping condition resulted in more peaked distributions than the peripheral visual condition (see Supplementary Figure), with the peak location (the mean of the von Mises distribution) broadly clustered around zero phase difference. Moreover, a separate model-free analysis of phase correspondence revealed highly similar results (see “Materials and Methods” Section and Supplementary Figure).

We investigated whether remapping responses showed any further specificity by analyzing the radial extent of the wedge stimulus’ remapping footprint. Using standard retinotopic mapping techniques with ring-shaped stimuli (Engel et al., 1994), the preferred eccentricity of each voxel in our above-defined stimulus-selective regions was estimated. Although this approach is not flawless in mapping out the most central regions of the visual field (Schira et al., 2009), it does allow for estimation of the phase specificity of responses to the wedge stimulus experiment at different eccentricities (see Figure 4J). The results of our analysis demonstrate that the remapped activation is stronger in the more central regions of early visual areas (Figure 4J). Because the more central retinotopic regions correspond to the higher-acuity regions surrounding the fovea, this is where information about the shape of retinal stimulation would be of most use to assist trans-saccadic integration of information. Note also that, due to cortical magnification, the extent of the perifoveal region on the foveal surface is at least five times the extent of the cortical region originally activated by the stimulus in the periphery in all subjects (for an example subject see Figures 4B,C). These findings suggest that remapping to regions near the fovea leads to appropriate scaling of the spatial extent of cortical activation, to compensate for cortical magnification between the periphery and fovea. This analysis also provides an important additional control for the possibility that activation resulting from visual stimulation in the periphery might constitute the source of the phase correspondence we find. If this were the case, phase correspondence would be greater in the more peripheral regions of the stimulus regions, as this would be the region where population receptive fields would be large enough to encompass both the central and peripheral stimulus locations on either side of the visual field. However, phase correspondence decreases with eccentricity for areas V1 through V3, indicating that peripheral stimulation cannot account for the remapping effects found in these areas. On the other hand, analysis of a higher-level region, V3A/B, shows greater effects in peripheral locations, consistent with either actual remapping or a possible ”spillover” due to peripheral stimulation. However, even here, it should be noted that responses of peripheral V3AB voxels were much greater in the remapping condition than in the peripheral stimulation condition. We conservatively excluded all higher visual regions from our analyses.

### Decoding Results

This standard phase-encoded design analysis shows strong similarities in voxels’ phase preferences in the Foveal Mapping conditions on the one hand, and the Remapping condition on the other. However, the lack of high-contrast visual stimulation to the foveal ROI in the Remapping condition caused signal amplitude to be far below the Foveal Mapping conditions’ signal levels—lowering the signal to noise ratio. We therefore decided to perform a multi-voxel pattern analysis (MVPA; Tong and Pratte, 2012) to gauge, from our foveal ROI, the instantaneous (per-TR) representation of remapped stimulus location. This MVPA analysis centers on creating a forward model of stimulus location (or, due to our periodic design, phase) from the Saccade-Mapping condition’s phase preference estimates, similar to earlier color-decoding studies (Brouwer and Heeger, 2009). This description is then used to decode the represented stimulus phase over time from the other conditions’ time-course data (see “Materials and Methods” Section). Importantly, this decoded stimulus phase time course can be normalized to discount differences in signal amplitude between conditions. This in turn allows us to focus on the location information represented in our ROI during a given condition, for each of the phases in our periodic stimulus presentation design. In parallel with the logic of our previous analyses, if location information is remapped, we should find periodic changes in decoded stimulus location, or phase, that correspond to the periodic changes due to visual stimulation.

**Figure 5:**
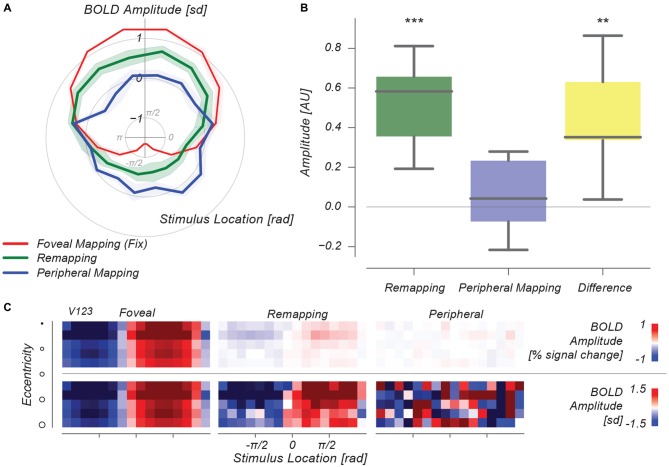
**Multivoxel pattern analysis (MVPA) results. (A)** Polar plot, depicting forward-model decoded BOLD amplitude per stimulus phase for three conditions. Decoded signals from the Foveal Mapping condition show a strong periodic signal varying across stimulus phase, whereas in the Peripheral Mapping condition no such signal exists. In the Remapping condition, the signal varies in phase with the signal from the Foveal Mapping condition, at intermediate amplitude.** (B)** The amplitude of this time-varying decoded stimulus-phase signal is significantly greater than both 0 and the Peripheral Mapping control condition. **(C)** Eccentricity-based representations of this decoded stimulus phase signal illustrate the difference in BOLD amplitude (expressed as % signal change) between conditions. **p < 0.01, ***p < 0.001.

Figure 5A shows these time-courses of decoded stimulus location represented in polar format. Strong changes in decoded stimulus location arise from direct foveal visual stimulation (red line), and no periodic changes in decoded stimulus location occur during our peripheral fixation control condition (blue line). In the remapping condition, however, there is a strong periodic signature in the time-course of decoded stimulus phase (green line). Importantly, this time-course is phase-aligned with the time-course of the foveal visual stimulation condition, confirming our remapping hypothesis. We quantified this similarity by linearly projecting the time-courses of the Remapping and Peripheral control conditions onto the Foveal visual stimulation condition’s time-course. These single-value measures are shown in Figure 5B. Only in the Remapping condition is the time-course significantly fluctuating in phase with the Foveal mapping condition (*p* < 0.001), and moreover, the difference between the Remapping and Peripheral mapping conditions is significant (*p* < 0.01). This analysis allows us to illustrate the role of signal amplitude differences between conditions. Figure 5C shows results as in Figure 5A for different eccentricity bins (*cf.,* 2j), with and without time-course normalization at bottom and top rows, respectively. It is evident that signal amplitude in the remapping case is strongly diminished in the Remapping condition, although the pattern of decoded stimulus phase is intact. This dissociation between MVPA-derived information and BOLD amplitude is reminiscent of earlier studies (Harrison and Tong, 2009). Normalizing these decoded time-courses reinstates the vigor of this pattern for the Remapping condition, whereas this operation highlights the absence of such a pattern in the Peripheral Mapping control condition.

These patterns from our phase preference and decoding analyses are unlikely to be due to errors in eye movements during the remapping condition runs. First, there were no differences in oculomotor behavior between the Foveal Mapping Saccades condition and Remapping condition. Second, we excluded from our analysis all runs in which the observers failed to fixate the appropriate fixation mark at any time around stimulus presentation, ensuring that Remapping condition signals were not caused by inadvertent visual stimulation. Third, our slow phase-encoding approach insulates the predictive remapping signal from the higher-frequency signals likely to be associated with small individual eye movement errors. Our results are also not due to residual screen persistence leading to visual input after the saccade landing, as our results did not depend on the luminance decay time of the projector (see “Materials and Methods” Section).

## Discussion

We report new evidence regarding the specific properties of remapping responses in visual cortex, focusing on remapping to foveal visual cortex, as remapping in this region has received little investigation. In previous studies (Merriam et al., 2003, 2007), activity was remapped to the expected target location after an eye movement, but these target locations were always in the periphery and the results did not permit a detailed evaluation of the spatial specificity of the remapped response. In our experiment, the expected target location encompassed foveal and parafoveal regions, the natural target of remapping for all goal-directed saccades. These regions have a large cortical representation of space that allowed us to determine the spatial profile of the remapping with greater precision. Critically, we showed that remapping activation in the foveal visual cortex reflects the retinotopic properties of the remapped stimulus, including its spatial orientation and spatial extent. The availability of this information is contingent on eye movements, as it is not present when observers merely attend to the same stimulus presented peripherally.

Evidence from both human imaging and monkey physiology studies suggests that remapping is strongest in higher visual areas located in the frontal and parietal lobes (Duhamel et al., 1992; Umeno and Goldberg, 1997; Merriam et al., 2003; Sommer and Wurtz, 2004; Heiser and Colby, 2006), and weaker at earlier levels of the visual hierarchy (Nakamura and Colby, 2002; Merriam et al., 2007). This pattern of results across the visual hierarchy is reminiscent of magnitude of covert attentional signals directed to discrete locations in the visual field, whose amplitude is also greater in parietal lobe (Silver et al., 2005, 2006). Previous imaging studies of remapping used an experimental design where observers made saccades across a salient target after its removal. These large lateral saccades caused cross-hemispheric remapping, allowing researchers to focus on more peripheral retinotopic locations as the targets of remapping responses (Merriam et al., 2003, 2007). Single-cell physiology investigations into remapping are dependent on the eccentricity of neurons that are encountered in an electrode traversal, and have commonly focused on more peripheral regions of the visual field (Nakamura and Colby, 2002). In everyday life, however, we make saccades towards items of interest instead of across them, implying that remapping responses for these targets should be found in the foveal representation of visual areas. Our findings indicate that remapping, seen previously with a shift in activation toward peripheral locations, also occurs when the item to be remapped is the saccade target itself, the typical case for the eye movements that we make most frequently in everyday life. The remapping effects found here demonstrate this predicted shift in activation patterns toward foveal representations of V1–V3. This conclusion is supported by our finding that remapping responses extended over the parafoveal regions of the retinotopic map, corresponding to the orientation and spatial extent that the stimulus would have had if its apex were fixated. Thus, the present study replicates and extends past results (Merriam et al., 2003, 2007).

The fovea is the location that the saccade target will occupy after the saccade, making it a very important region in the retinotopic map. Recent monkey electrophysiology results have suggested that presaccadic receptive field shifts in FEF occur predominantly in the direction of the saccade target (Zirnsak et al., 2014). A study of area V4 also found this receptive field shift toward the saccade target but only for later activity. Early activity showed the remapping shifts similar to those originally reported by Duhamel et al. (1992) and others. In our study of occipital cortex, we could not inspect the retinotopic region of the saccade target, as our study was designed to have strong visual stimulation in that location in the Remapping condition. This makes it impossible to separate visual and oculomotor signals due to the fact that BOLD signals are spatially and temporally low-pass when compared to single-unit recordings. Further studies are needed to distinguish between different models of trans-saccadic integration, and their predominance when assessed in different brain regions and measurement modalities.

Stimulus category can be decoded from foveal, unstimulated regions of V1, when observers attended to the object shape of peripheral stimuli (Williams et al., 2008). These authors ruled out a role of eye movement behavior in their findings, whereas in our study eye movements are a necessary condition for the remapping responses in unstimulated regions to occur. This difference between the respective studies could be due to the differences in task or stimuli (color task on wedge stimuli vs. object comparison task on synthetic object stimuli). Another key difference was that in the present study, we tested the similarity of activity patterns for stimuli presented in the fovea and in the periphery under conditions of remapping.

What information do remapping responses carry, and how might this support the visual stability of object representations (Wurtz, 2008; Cavanagh et al., 2010)? Psychophysical studies have reported conflicting evidence regarding whether feature information is transmitted around the time of an eye movement (Melcher, 2005; Burr et al., 2007; Knapen et al., 2009; Bruno et al., 2010; Knapen et al., 2010). From the present results, it is not possible to conclude whether the remapped signals that we find in visual cortex should be construed as part of an object- or scene-based representation, or simply the transmission of spatial information. An fMRI investigation into the remapping of object- or scene-based representations would require a different set of stimuli to focus on responses in higher-level visual areas (Golomb and Kanwisher, 2012). We found that peripheral voxels in visual areas higher than V3 may respond to such a large region of retinotopic space, leading to considerable overlap between pre- and post-saccadic retinotopic locations (see Supplementary Figure), even with saccade lengths of 14° of the present study. Adapting our paradigm to higher-level vision would be challenging, because saccades would have to be large enough to prohibit pre- and post-saccadic incidence of stimuli in the same receptive fields, which are much larger at higher levels of the visual hierarchy (Dumoulin and Wandell, 2008).

It is interesting to consider why a stimulus that has disappeared from the screen should elicit a remapping response at all. The present study shares this issue with previous fMRI investigations of perisaccadic remapping where the stimulus was removed about 250 ms before saccade onset; these previous studies also found activation at the remapped location (Merriam et al., 2003, 2007). There are two factors, one oculomotor and one visual, that suggest that there still should be remapping for stimuli that have disappeared within a brief interval before the saccade. First, the remapping must be set in motion well before the saccade lands at its target, and even before the saccade launches. Research suggests that about 80 ms prior to saccade onset, the landing has been programmed and can no longer be changed (saccadic “dead time”; Findlay and Harris, 1984; Ludwig et al., 2007). The remaining 250 ms to be bridged in our experimental design can be accounted for by visual persistence. The time window for the temporally extended visual representation of stimuli, such as visual persistence and iconic memory, lasts for up to 250 ms (Coltheart, 1980; Nikolić et al., 2009), and some (amodal) representations of an occluded stimulus continue even longer (Joseph and Nakayama, 1999). Apparent motion can be perceived between stimuli with temporal gaps of 250 ms or more, suggesting that the visual system maintains representations of targets for some time after their disappearance, and a recent study demonstrates that these representations are remapped (Szinte and Cavanagh, 2011). Thus, there is sufficient opportunity for interactions between the persistent neural representations of a stimulus and the corrollary discharge of the saccade that will bring that stimulus to the fovea, if the time between stimulus and saccade is 250 ms.

It is clear from behavioral results that predictive remapping is spatially localized (Collins et al., 2009; Rolfs et al., 2010; Szinte and Cavanagh, 2011). Our present results provide a neural correlate of the spatial profile of the remapped response, showing that information about the shape of retinal stimulation of the peripheral saccade target is transmitted, with appropriate rescaling, to the fovea around the time that the eyes move. Such a process is a prime candidate for the neural underpinning of our sense of world-centered location in the face of continuing eye movements.

## Author Contributions

TK, JDS, FT and PC conceived research, TK and JDS performed experiments, TK analyzed the data and TK, JDS, FT and PC wrote the article.

## Funding

This work was supported by a National Eye Institute (NEI) grant (R01 EY017082) and Vanderbilt University Discovery grant to FT, NEI fellowship F32 EY019448 to JS and an Agence Nationale de la Recherche (ANR) Chaire d’Excellence and funding from the European Research Council under the European Union’s Seventh Framework Programme (FP7/2007-2013)/ERC grant agreement n° AG 324070 to PC. TK was supported by Nederlandse Organisatie voor Wetenschappelijk Onderzoek (NWO) VENI and ORA (451-09-016 and 464-11-030, respectively).

## Conflict of Interest Statement

The authors declare that the research was conducted in the absence of any commercial or financial relationships that could be construed as a potential conflict of interest.

## References

[B1] Brouwer G. J. Heeger D. J. (2009). Decoding and reconstructing color from responses in human visual cortex. J. Neurosci. 29, 13992–14003. 10.1523/JNEUROSCI.3577-09.2009 19890009 PMC2799419

[B2] Bruno A. Ayhan I. Johnston A. (2010). Retinotopic adaptation-based visual duration compression. J. Vis. 10:30. 10.1167/10.10.30 20884495

[B3] Burr D. C. Tozzi A. Morrone M. C. (2007). Neural mechanisms for timing visual events are spatially selective in real-world coordinates. Nat. Neurosci. 10, 423–425. 10.1038/nn1874 17369824

[B4] Buswell G. T. (1935). How People Look at Pictures. Chicago, IL: University of Chicago Press.

[B5] Cavanagh P. Hunt A. R. Afraz A. Rolfs M. (2010). Visual stability based on remapping of attention pointers. Trends Cogn. Sci. 14, 147–153. 10.1016/j.tics.2010.01.007 20189870 PMC2847621

[B6] Collins T. Rolfs M. Deubel H. Cavanagh P. (2009). Post-saccadic location judgments reveal remapping of saccade targets to non-foveal locations. J. Vis. 9, 29.1–29.9. 10.1167/9.5.29 19757907

[B7] Coltheart M. (1980). Iconic memory and visible persistence. Percept. Psychophys. 27, 183–228. 10.3758/bf03204258 6992093

[B8] Connolly J. D. Vuong Q. C. Thiele A. (2015). Gaze-dependent topography in human posterior parietal cortex. Cereb. Cortex 25, 1519–1526. 10.1093/cercor/bht344 24351977 PMC4428299

[B9] Deubel H. Schneider W. X. (1996). Saccade target selection and object recognition: evidence for a common attentional mechanism. Vis. Res. 36, 1827–1837. 10.1016/0042-6989(95)00294-4 8759451

[B10] Duhamel J.-R. Colby C. L. Goldberg M. E. (1992). The updating of the representation of visual space in parietal cortex by intended eye movements. Science 255, 90–92. 10.1126/science.1553535 1553535

[B11] Dumoulin S. O. Wandell B. A. (2008). Population receptive field estimates in human visual cortex. Neuroimage 39, 647–660. 10.1016/j.neuroimage.2007.09.034 17977024 PMC3073038

[B12] Engel S. A. Rumelhart D. E. Wandell B. A. Lee A. T. Glover G. H. Chichilnisky E. J.. (1994). fMRI of human visual cortex. Nature 369:525. 10.1038/369525a0 8031403

[B13] Findlay J. M. Harris L. R. (1984). “Small saccades to double-stepped targets moving in two dimensions,” in Theoretical and Applied Aspects of Eye Movement Research, eds Gale A. G. Johnson F. (Amsterdam: Elsevier), 71–78.

[B14] Golomb J. D. Kanwisher N. (2012). Higher level visual cortex represents retinotopic, not spatiotopic, object location. Cereb. Cortex 22, 2794–2810. 10.1093/cercor/bhr357 22190434 PMC3491766

[B15] Harrison S. A. Tong F. (2009). Decoding reveals the contents of visual working memory in early visual areas. Nature 458, 632–635. 10.1038/nature07832 19225460 PMC2709809

[B16] Heiser L. M. Colby C. L. (2006). Spatial updating in area LIP is independent of saccade direction. J. Neurophysiol. 95, 2751–2767. 10.1152/jn.00054.2005 16291805

[B17] Joseph J. S. Nakayama K. (1999). Amodal representation depends on the object seen before partial occlusion. Vis. Res. 39, 283–292. 10.1016/s0042-6989(98)00065-0 10326136

[B18] Knapen T. Rolfs M. Cavanagh P. (2009). The coordinate system of the motion aftereffect is retinotopic. J. Vis. 9:682 10.1167/9.8.682 19757894

[B19] Knapen T. Rolfs M. Wexler M. Cavanagh P. (2010). The reference frame of the tilt aftereffect. J. Vis. 10, 8.1–8.13. 10.1167/10.1.8 20143901

[B20] Larsson J. Heeger D. J. (2006). Two retinotopic visual areas in human lateral occipital cortex. J. Neurosci. 26, 13128–13142. 10.1523/JNEUROSCI.1657-06.2006 17182764 PMC1904390

[B21] Ludwig C. J. H. Mildinhall J. W. Gilchrist I. D. (2007). A population coding account for systematic variation in saccadic dead time. J. Neurophysiol. 97, 795–805. 10.1152/jn.00652.2006 17108094

[B22] Melcher D. (2005). Spatiotopic transfer of visual-form adaptation across saccadic eye movements. Curr. Biol. 15, 1745–1748. 10.1016/j.cub.2005.08.044 16213821

[B23] Merriam E. P. Gardner J. L. Movshon J. A. Heeger D. J. (2013). Modulation of visual responses by gaze direction in human visual cortex. J. Neurosci. 33, 9879–9889. 10.1523/jneurosci.0500-12.2013 23761883 PMC3682387

[B24] Merriam E. P. Genovese C. R. Colby C. L. (2003). Spatial updating in human parietal cortex. Neuron 39, 361–373. 10.1016/s0896-6273(03)00393-3 12873391

[B25] Merriam E. P. Genovese C. R. Colby C. L. (2007). Remapping in human visual cortex. J. Neurophysiol. 97, 1738–1755. 10.1152/jn.00189.2006 17093130 PMC2292409

[B26] Nakamura K. Colby C. L. (2002). Updating of the visual representation in monkey striate and extrastriate cortex during saccades. Proc. Natl. Acad. Sci. U S A 99, 4026–4031. 10.1073/pnas.052379899 11904446 PMC122642

[B27] Neupane S. Guitton D. Pack C. C. (2016). Two distinct types of remapping in primate cortical area V4. Nat. Commun. 7:10402. 10.1038/ncomms10402 26832423 PMC4740356

[B28] Nikolić D. Häusler S. Singer W. Maass W. (2009). Distributed fading memory for stimulus properties in the primary visual cortex. PLoS Biol. 7:e1000260. 10.1371/journal.pbio.1000260 20027205 PMC2785877

[B29] Rolfs M. Jonikaitis D. Deubel H. Cavanagh P. (2010). Predictive remapping of attention across eye movements. Nat. Neurosci. 14, 252–256. 10.1038/nn.2711 21186360

[B30] Schira M. M. Tyler C. W. Breakspear M. Spehar B. (2009). The foveal confluence in human visual cortex. J. Neurosci. 29, 9050–9058. 10.1523/JNEUROSCI.1760-09.2009 19605642 PMC6665445

[B31] Sereno M. I. Dale A. M. Reppas J. B. Kwong K. K. Belliveau J. W. Brady T. J.. (1995). Borders of multiple visual areas in humans revealed by functional magnetic resonance imaging. Science 268, 889–893. 10.1126/science.7754376 7754376

[B32] Sereno M. I. McDonald C. T. Allman J. M. (1994). Analysis of retinotopic maps in extrastriate cortex. Cereb. Cortex 4, 601–620. 10.1093/cercor/4.6.601 7703687

[B33] Silver M. A. Ress D. Heeger D. J. (2005). Topographic maps of visual spatial attention in human parietal cortex. J. Neurophysiol. 94, 1358–1371. 10.1152/jn.01316.2004 15817643 PMC2367310

[B34] Silver M. A. Ress D. Heeger D. J. (2006). Neural correlates of sustained spatial attention in human early visual cortex. J. Neurophysiol. 97, 229–237. 10.1152/jn.00677.2006 16971677 PMC1868502

[B35] Sommer M. A. Wurtz R. H. (2004). What the brain stem tells the frontal cortex. II. Role of the SC-MD-FEF pathway in corollary discharge. J. Neurophysiol. 91, 1403–1423. 10.1152/jn.00740.2003 14573557

[B36] Strappini F. Pitzalis S. Snyder A. Z. McAvoy M. P. Sereno M. I. Corbetta M.. (2015). Eye position modulates retinotopic responses in early visual areas: a bias for the straight-ahead direction. Brain Struct. Funct. 220, 2587–2601. 10.1007/s00429-014-0808-7 24942135 PMC4549389

[B37] Swisher J. D. Halko M. A. Merabet L. B. McMains S. A. Somers D. C. (2007). Visual topography of human intraparietal sulcus. J. Neurosci. 27, 5326–5337. 10.1523/JNEUROSCI.0991-07.2007 17507555 PMC6672354

[B38] Szinte M. Cavanagh P. (2011). Spatiotopic apparent motion reveals local variations in space constancy. J. Vis. 11:4. 10.1167/11.2.4 21304172

[B39] Tong F. Pratte M. S. (2012). Decoding patterns of human brain activity. Annu. Rev. Psychol. 63, 483–509. 10.1146/annurev-psych-120710-100412 21943172 PMC7869795

[B40] Umeno M. M. Goldberg M. E. (1997). Spatial processing in the monkey frontal eye field. I. Predictive visual responses. J. Neurophysiol. 78, 1373–1383. 9310428 10.1152/jn.1997.78.3.1373

[B41] Williams M. A. Baker C. I. Op de Beeck H. P. Shim W. M. Dang S. Triantafyllou C.. (2008). Feedback of visual object information to foveal retinotopic cortex. Nat. Neurosci. 11, 1439–1445. 10.1038/nn.2218 18978780 PMC2789292

[B42] Wurtz R. H. (2008). Neuronal mechanisms of visual stability. Vis. Res. 48, 2070–2089. 10.1016/j.visres.2008.03.021 18513781 PMC2556215

[B43] Zirnsak M. Steinmetz N. A. Noudoost B. Xu K. Z. Moore T. (2014). Visual space is compressed in prefrontal cortex before eye movements. Nature 507, 504–507. 10.1038/nature13149 24670771 PMC4064801

