# Supplementary material for: Oculomotor Remapping of Visual Information to Foveal Retinotopic Cortex
Source: Front Syst Neurosci. 2016 Jun 21;10:54. doi: 10.3389/fnsys.2016.00054 (PMC4915294; doi:10.3389/fnsys.2016.00054)

*Figure S1.* ***a.*** *Fit parameters of von Mises distributions when allowing both parameters to vary. Data from V1-V3, colors as in figures 1,2,3. 50 Voxels were randomly chosen from each area and pooled across areas, and fitting was performed on these pooled voxels. 1000 iterations of this procedure were performed per subject. Although the mean parameter that describes the position of the peak of the distribution varies for the remapping condition its distribution is centered around 0 (-0.06, standard deviation 0.85 rad).* ***b.*** *We constructed a model-free measure of phase correspondence by taking the absolute value of the phase difference between remapping and saccade-foveal mapping conditions, and subtracting the absolute value of the phase difference between peripheral mapping and saccade-foveal mapping conditions. This resulting value is divided by pi/2 to deliver a value of 1 for perfect shape correspondence. Values greater than 0 indicate remapping of shape information. These results are shown for each of the 7 subjects separately. (yellow, Vanderbilt, green, UvA, gray, average). These data show a similar trend as the fit parameters in figure 4g-i. $p < 0.01$ for all areas, t-test per area.* ***c.*** *Control analysis for eye movement errors. The above value, plotted per visual area and subject for all runs with total fixation error > median vs all runs with total fixation error < median. There is no difference between the remapping signals in these different runs (see histogram), and we conclude that eye movement errors did not impact our results. Testing (t-test across subjects) for each area separately, all $p > 0.4$* ***d.*** *Dependence of remapping responses on eccentricity, cf Figure 4j. Remapping signature in V3 is stronger in the foveal regions of the retinotopic map.* ***e.*** *For V3AB, however, the phase correspondence measure is greater for more peripheral regions of the visual field.* ***f.*** *Voxel phase preferences in the remapping and peripheral mapping signals relative to the fixation foveal mapping instead of the saccade foveal mapping condition, cf. Figure 2d-f.*

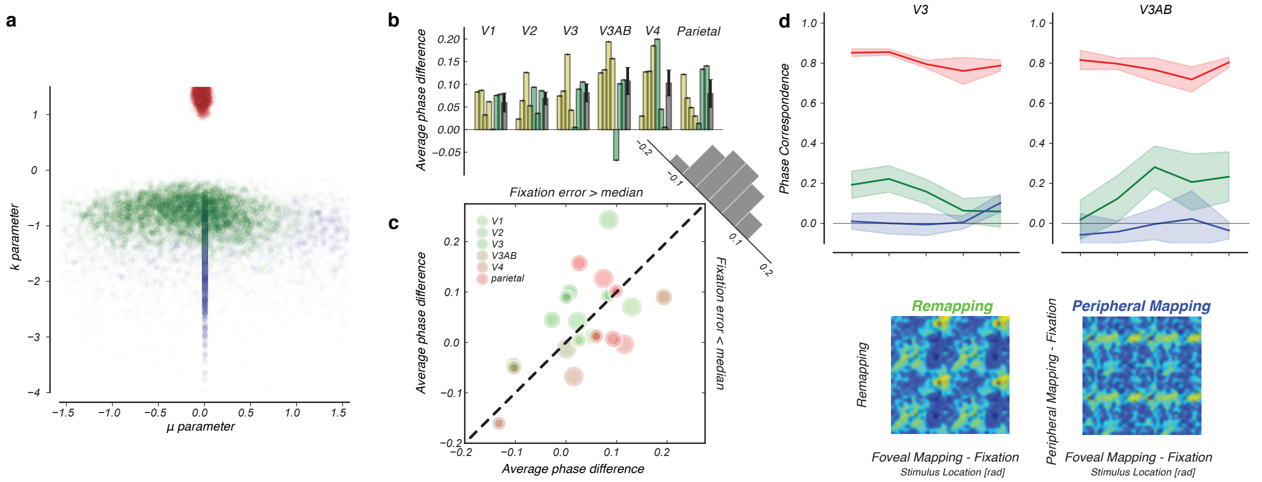

Supplement: Supplementary file 1 [file Image_1.pdf]
